# Supplementary material for: Longitudinal path analysis for the directional association of depression, anxiety and posttraumatic stress disorder with their comorbidities and associated factors among postpartum women in Northwest Ethiopia: A cross-lagged autoregressive modelling study
Source: PLoS One. 2022 Aug 15;17(8):e0273176. doi: 10.1371/journal.pone.0273176 (PMC9377604; doi:10.1371/journal.pone.0273176)

PLOS ONE

Longitudinal path analysis of the relation between comorbidity of anxiety, depression and posttraumatic stress disorder among postpartum women with and without maternal morbidities in Northwest Ethiopia: A cross-lagged autoregressive analysis

--Manuscript Draft--

Manuscript Number: PONE-D-22-04381

Article Type: Research Article

Full Title: Longitudinal path analysis of the relation between comorbidity of anxiety, depression and posttraumatic stress disorder among postpartum women with and without maternal morbidities in Northwest Ethiopia: A cross-lagged autoregressive analysis

Short Title: Path analysis of the relation between comorbidity of anxiety, depression and posttraumatic stress disorder

Corresponding Author: Marelign Tilahun Malaju, MPH in Epidemiology and Biostatistics
Debre Tabor University
Debre Tabor, Amhara ETHIOPIA

Keywords: Longitudinal, Path analysis, Comorbidity, Cross-lagged autoregressive analysis, Depression, Anxiety, PTSD

Abstract:

Abstract

Introduction: Vulnerability for depression, anxiety and posttraumatic stress disorder symptoms increases during the postpartum period as it is a time where mothers can undergo significant changes. However, the direct and indirect factors associated with comorbidity of depression, anxiety and PTSD symptoms is not well investigated in Ethiopia

Objective: The aim of this study was to determine the direct and indirect factors associated with longitudinal comorbidity of depression, anxiety and PTSD symptoms following childbirth.

Methods: A total of 775 women consented to participate at the first, second and third follow-up of the study (6th, 12th and 18th week of postpartum period) during October, 2020 – March, 2021.

Women were recruited after c[illegible]irth and before discharge using the WHO maternal morbidity criteria to identify exposed and non-exposed groups. A cr[illegible]ss-lagged autoregressive pa[illegible] analysis was carried out using Stata version 16 software in order to determine the autoregressive effects and direct and indirect effects of variables on the outcome variables.

Results: A total of 775 wo[illegible]men participated at the first, second and third follow-up of the study. Comorbidity of anxiety with depression (14.5%, 12.1% and 8.1% at the 6th, 12th and 18th week of postpartum period respectively) was the most common.

The coexistence of PTSD with depression (6.4%, 6.6% and 3.0%), PTSD with anxiety (9.3%, 6.5% and 3.0%) and triple comorbidity (9.3%, 6.5% and 3.0%) were equally prevalent throughout the follow up period. Moreover, anxiety was found to be a causal risk factor for comorbidity of anxiety with depression, comorbidity of depression with PTSD and for triple comorbidity.

Direct maternal morbidity, fear of childbirth, higher gravidity, perceived traumatic childbirth and indirect maternal morbidity were found to have a direct and indirect positive association with comorbidities of depression, anxiety and PTSD. In contrast, higher parity, higher family size and higher social support have a direct and indirect negative association.

Conclusion: Postnatal mental health screening, early diagnosis and treatment of maternal morbidities, developing encouraging strategies for social support and providing adequate information about birth procedures and response to mothers' needs during childbirth are essential to avert comorbidity of anxiety, depression and PTSD in the postpartum period.

Order of Authors:

Marelign Tilahun Malaju, MPH in Epidemiology and Biostatistics

Getu Degu Alene, PhD

Telake Azale Bisetegn, PhD

Powered by Editorial Manager® and ProduXion Manager® from Aries Systems Corporation

Additional Information:	
**Question**	**Response**
**Financial Disclosure**<br><br>Enter a financial disclosure statement that describes the sources of funding for the work included in this submission. Review the submission guidelines for detailed requirements. View published research articles from *PLOS ONE* for specific examples.<br><br>This statement is required for submission and **will appear in the published article** if the submission is accepted. Please make sure it is accurate.<br><br>**Unfunded studies**<br>Enter: *The author(s) received no specific funding for this work.*<br><br>**Funded studies**<br>Enter a statement with the following details:<br>• Initials of the authors who received each award<br>• Grant numbers awarded to each author<br>• The full name of each funder<br>• URL of each funder website<br>• Did the sponsors or funders play any role in the study design, data collection and analysis, decision to publish, or preparation of the manuscript?<br>• **NO** - Include this sentence at the end of your statement: *The funders had no role in study design, data collection and analysis, decision to publish, or preparation of the manuscript.*<br>• **YES** - Specify the role(s) played.<br><br>* typeset	The author(s) received no specific funding for this work.
**Competing Interests**<br><br>Use the instructions below to enter a competing interest statement for this submission. On behalf of all authors, disclose any competing interests that could be perceived to bias this work—acknowledging all financial support and any other relevant financial or non-financial competing interests.	The authors have declared that no competing interests exist.

Powered by Editorial Manager® and ProduXion Manager® from Aries Systems Corporation

This statement is required for submission and **will appear in the published article** if the submission is accepted. Please make sure it is accurate and that any funding sources listed in your Funding Information later in the submission form are also declared in your Financial Disclosure statement.<br><br>View published research articles from *PLOS ONE* for specific examples.<br><br>**NO authors have competing interests**<br><br>Enter: *The authors have declared that no competing interests exist.*<br><br>**Authors with competing interests**<br><br>Enter competing interest details beginning with this statement:<br><br>*I have read the journal's policy and the authors of this manuscript have the following competing interests: [insert competing interests here]*<br><br>* typeset	
**Ethics Statement**<br><br>Enter an ethics statement for this submission. This statement is required if the study involved:<br><br>• Human participants<br>• Human specimens or tissue<br>• Vertebrate animals or cephalopods<br>• Vertebrate embryos or tissues<br>• Field research<br><br>Write "N/A" if the submission does not require an ethics statement.<br><br>General guidance is provided below. Consult the submission guidelines for detailed instructions. **Make sure that all information entered here is included in the**	Ethical approval was obtained from Institutional Review Board of Bahir Dar University (Reference number: 00225/2020)

**Methods section of the manuscript.**<br><br>**Format for specific study types**<br><br>**Human Subject Research (involving human participants and/or tissue)**<br>• Give the name of the institutional review board or ethics committee that approved the study<br>• Include the approval number and/or a statement indicating approval of this research<br>• Indicate the form of consent obtained (written/oral) or the reason that consent was not obtained (e.g. the data were analyzed anonymously)<br><br>**Animal Research (involving vertebrate animals, embryos or tissues)**<br>• Provide the name of the Institutional Animal Care and Use Committee (IACUC) or other relevant ethics board that reviewed the study protocol, and indicate whether they approved this research or granted a formal waiver of ethical approval<br>• Include an approval number if one was obtained<br>• If the study involved *non-human primates*, add *additional details* about animal welfare and steps taken to ameliorate suffering<br>• If anesthesia, euthanasia, or any kind of animal sacrifice is part of the study, include briefly which substances and/or methods were applied<br><br>**Field Research**<br><br>Include the following details if this study involves the collection of plant, animal, or other materials from a natural setting:<br>• Field permit number<br>• Name of the institution or relevant body that granted permission	
**Data Availability**<br><br>Authors are required to make all data underlying the findings described fully available, without restriction, and from the time of publication. PLOS allows rare exceptions to address legal and ethical concerns. See the PLOS Data Policy and FAQ for detailed information.	Yes - all data are fully available without restriction

Powered by Editorial Manager® and ProduXion Manager® from Aries Systems Corporation

A Data Availability Statement describing where the data can be found is required at submission. Your answers to this question constitute the Data Availability Statement and **will be published in the article**, if accepted.<br><br>**Important:** Stating 'data available on request from the author' is not sufficient. If your data are only available upon request, select 'No' for the first question and explain your exceptional situation in the text box.<br><br>Do the authors confirm that all data underlying the findings described in their manuscript are fully available without restriction?	
**Describe where the data may be found in full sentences. If you are copying our sample text, replace any instances of XXX with the appropriate details.**<br><br>• If the data are **held or will be held in a public repository**, include URLs, accession numbers or DOIs. If this information will only be available after acceptance, indicate this by ticking the box below. For example: *All XXX files are available from the XXX database (accession number(s) XXX, XXX.*).<br>• If the data are all contained **within the manuscript and/or Supporting Information files**, enter the following: *All relevant data are within the manuscript and its Supporting Information files.*<br>• If neither of these applies but you are able to provide **details of access elsewhere**, with or without limitations, please do so. For example:<br><br>*Data cannot be shared publicly because of [XXX]. Data are available from the XXX Institutional Data Access / Ethics Committee (contact via XXX) for researchers who meet the criteria for access to confidential data.*<br><br>*The data underlying the results*	All relevant data are within the manuscript and its Supporting Information files.

presented in the study are available from (include the name of the third party and contact information or URL).<br>• This text is appropriate if the data are owned by a third party and authors do not have permission to share the data.<br><br>* typeset	
Additional data availability information:	Tick here if the URLs/accession numbers/DOIs will be available only after acceptance of the manuscript for publication so that we can ensure their inclusion before publication.

Powered by Editorial Manager® and ProduXion Manager® from Aries Systems Corporation

Manuscript

Click here to access/download;Manuscript;Multimorbidity PLoSONE. Dep,Anxiety,PTSD.docx

Longitudinal path analysis of the relation between comorbidity of anxiety, depression and posttraumatic stress disorder among postpartum women with and without maternal morbidities in Northwest Ethiopia: A cross-lagged autoregressive analysis

Marelign Tilahun Malaju*[1,2,] Getu Degu Alene[2], Telake Azale Bisetegn [3]

[1]Department of Public Health, College of Health Sciences, Debre Tabor University, Debre Tabor, Ethiopia

[2]School of Public Health, College of Medicine and Health Sciences, Bahir Dar University, Bahir Dar, Ethiopia

[3]Department of Health Education and Behavioral Sciences, College of Medicine and Health Sciences, University of Gondar, Gondar, Ethiopia

*Corresponding author

Email: marikum74@gmail.com

Abstract

Introduction: Vulnerability for depression, anxiety and posttraumatic stress disorder symptoms increases during the postpartum period as it is a time where mothers can undergo significant changes. However, the direct and indirect factors associated with comorbidity of depression, anxiety and PTSD symptoms is not well investigated in Ethiopia

Objective: The aim of this study was to determine the direct and indirect factors associated with longitudinal comorbidity of depression, anxiety and PTSD symptoms following childbirth.

Methods: A total of 775 women consented to participate at the first, second and third follow-up of the study (6th, 12th and 18th week of postpartum period) during October, 2020 – March, 2021. Women were recruited after childbirth and before discharge using the WHO maternal morbidity criteria to identify exposed and non-exposed groups. A cross-lagged autoregressive path analysis was carried out using Stata version 16 software in order to determine the autoregressive effects and direct and indirect effects of variables on the outcome variables.

Results: A total of 775 women participated at the first, second and third follow-up of the study. Comorbidity of anxiety with depression (14.5%, 12.1% and 8.1% at the 6th, 12th and 18th week of postpartum period respectively) was the most common. The coexistence of PTSD with depression (6.4%, 6.6% and 3.0%), PTSD with anxiety (9.3%, 6.5% and 3.0%) and triple comorbidity (9.3%, 6.5% and 3.0%) were equally prevalent throughout the follow up period. Moreover, anxiety was found to be a causal risk factor for comorbidity of anxiety with depression, comorbidity of depression with PTSD and for triple comorbidity.

Direct maternal morbidity, fear of childbirth, higher gravidity, perceived traumatic childbirth and indirect maternal morbidity were found to have a direct and indirect positive association with comorbidities of depression, anxiety and PTSD. In contrast, higher parity, higher family size and higher social support have a direct and indirect negative association.

Conclusion: Postnatal mental health screening, early diagnosis and treatment of maternal morbidities, developing encouraging strategies for social support and providing adequate information about birth procedures and response to mothers' needs during childbirth are essential to avert comorbidity of anxiety, depression and PTSD in the postpartum period.

Key words: Longitudinal, Path analysis, Comorbidity, Cross-lagged autoregressive analysis, Depression, Anxiety, PTSD

Introduction

Vulnerability for depression, anxiety and posttraumatic stress disorder symptoms increases during the postpartum period as it is a time where mothers can undergo significant changes (1, 2). While depression is a state of low mood or loss of pleasure or interest in activities, anxiety is generally characterized by feelings of tension, worried thoughts and physical changes(3). Whereas, post-traumatic stress disorder (PTSD) refers to a cluster of psychological symptoms that develop following exposure to a severe stressor or traumatic event associated with a real or perceived threat of death or threat to physical integrity of the person or others. Symptom clusters involve re-experiencing the event (intrusion symptoms), persistent avoidance of stimuli associated with the event (avoidance symptoms), negative mood alterations, and increased arousal and reactivity(3).

In low and middle-income countries (LMICs), the prevalence rates of postnatal depression ranged between 10 to 20%(4). The global estimates of anxiety prevalence rates during the postnatal period were about 8-10% (5). Prevalence rate of depression was reported to be 18.3% during the postnatal periods in a systematic review, whereas for postnatal anxiety it was estimated to be 14%(6). Higher prevalence rates were found in other studies. For example, in a study from Cape Town, 34.7% of postnatal women were found to have depression symptoms (7). Another study from Uganda also reported that 43% of the participants were found to have postpartum depressive symptoms(8). For postnatal anxiety, a prevalence rate of 17.1% was reported by a systematic review (9). A prevalence rate of 13.7% was also reported by another meta-analysis during the postpartum period for anxiety symptoms (10). The postnatal anxiety prevalence rate was found to be 14% in a review of studies from Africa (6).

There is also a growing body of literature suggesting that women may perceive childbirth as traumatic and develop PTSD as a result of a traumatic birth (11-13). Traumatic birth has been defined as an event occurring during labour and birth that may be a serious threat to the life and safety of the mother and/or child(14). Literatures have reported that 9 to 45.5% of mothers perceived childbirth as traumatic (11, 15, 16) and estimates of post-traumatic stress disorder in the postpartum period were reported to be 3-15% in previous literatures (17-20). A meta-analysis reported that 4% of women were found to have PTSD symptoms in the postpartum period and this increases to 18.5% in women with complications in pregnancy (20). A study from Norway among postpartum women in reported that 1.8% of women had PTSD following childbirth (21), whereas in Iran it has been found that 20% of women had PTSD after childbirth (22).

PTSD is not the result of a single cause (i.e., a traumatic stressor), but is the consequence of various interacting variables(13). It is the outcome of the interplay between antepartum vulnerability factors, the events during delivery and postpartum factors that interact over time during perinatal period (18). Childbirth related PTSD usually occurs as a result of complications during pregnancy or birth (17, 23). However, it may also be a continuation of pre-existing PTSD, a reactivation of PTSD triggered by childbirth related events that had previously resolved or new-onset PTSD in response to an event which is not related to childbirth(12, 24, 25).

The comorbidity of depression and anxiety symptoms was found to be 13.1% during the first 8 weeks postpartum(26). Another study has also reported comorbidity rates of 18.8% and 8.9% at 4-6 weeks and 6 months postpartum respectively(27). Comorbidity of PTSD with other psychiatric disorders has been also reported. While its co-morbidity rate with depression was found to be 10.1% and 4.9% at 4-6 weeks and 6 months of postpartum period, it is reported that there is a comorbidity rate of 7.2% and 5.4% with anxiety during the same period(27). The rates of multimorbidity after childbirth were rarely investigated and found to be 6.6% at 4–6 weeks and 3.4% at 6 months postpartum in one study(27). A systematic review also reported 2-3% prevalence rate of multimorbidity after childbirth (28).

While the co-morbidity of depression with anxiety, depression with PTSD and anxiety with PTSD has been investigated, the co-existence of all the three disorders (depression, anxiety and PTSD) in the postpartum period is not well investigated. In addition, previous studies were cross-sectional, providing limited insights into longitudinal patterns of comorbid depression, anxiety and PTSD and its predictors during postpartum period. In Ethiopia, no study investigating the multimorbidity in the postpartum period has been identifie[illegible]nderstanding if triple co-morbidity occurs and establishing its prevalence is important, as women may present with complex and mixed symptoms, making them hard to identify and treat. Therefore, the aim of this study was to determine the direct and indirect factors associated with longitudinal comorbidity of depression, anxiety and PTSD following childbirth.

Methods

Study design and Study Area

As part of the health facility linked community based prospective follow-up study conducted in Northwest Ethiopia to determine the effect of maternal morbidities on maternal health related quality of life, functional status mental health problems, postpartum women were recruited in four

hospitals of south Gondar zone, Northwest Ethiopia. The data collection took place between October 1, 2020 and March 30, 2021. South Gondar is located at 650 km Northwest from Addis Ababa the capital city of Ethiopia.

Study population

A total of 775 women consented to participate in the study and participated at the first, second and third follow-up of the study (6th, 12th and 18th week of postpartum period). Recruitment of the study participants was done after child birth and before the time of discharge. Women with any of the direct maternal morbidities were recruited into the exposed group and those without the direct maternal morbidities were into the non-exposed group based on the WHO maternal morbidity criteria(29).

Eligibility/Inclusion Criteria

Women aged 15 years and above, with preterm, term or post term delivery and with live birth, still birth or fetal death were included in the study. The PTSD criterion A was not considered as an exclusion criterion, because childbirth related negative events and emotions that do not satisfy the criterion A can cause symptoms that could qualify as a PTSD diagnosis(13).

Sampling procedure

All exposed women with direct maternal morbidity included in the study and non-exposed women without direct maternal morbidities were selected by simple random sampling method using their chart number on daily bases. With 1:2 ratio of exposed to non-exposed mothers, this recruitment procedure continued prospectively until the required sample size was fulfilled. Women were asked for consent to participate in the study and after getting their consent and full address, appointments were made at their home to collect the data for the follow up study.

Outcome and Independent variables

Comorbidity of depression, anxiety and posttraumatic stress disorder was taken as the outcome variable. Direct maternal morbidities(obstetric hemorrhage, hypertensive disorders, obstructed labour, puerperal sepsis, gestational diabetes mellitus, perineal tear), indirect maternal morbidities (anemia, malaria, hypertension, asthma, tuberculosis, HIV), socio-demographic variables (age, educational status, marital status, religion, ethnicity, occupation, monthly expenditure), residence, obstetric variables (parity, mode of delivery, gestational age at birth, birth weight, birth interval, fetal death, unwanted pregnancies, antenatal care visit, history of abortion) and psychosocial factors (social support and fear of child birth) were taken as the main independent variables.

Measures of variables

Depression, Anxiety and Stress

The short version of depression, anxiety and stress scale 21 (DASS-21) questionnaire was used to measure depression, anxiety and stress. The instrument has 21 items with three domains. Each domain comprises seven items assessing symptoms of depression, anxiety and stress. In this study a score ≥ 10 was considered for a mother to have a symptom of depression. A cut-off score of ≥ 8 was considered to have symptoms of anxiety and a score of ≥ 15 was considered to have symptoms of stress. This instrument was used previously in Ethiopia (30, 31).

Posttraumatic Stress Disorder

The childbirth stressor was operationalized by using the Traumatic Event Scale (TES)(32, 33). In this scale, the items concerning criterion A (stressor) were formulated as follows:

1. “The childbirth was a trying experience.”
2. “The childbirth was a threat to my physical integrity.”
3. “During the childbirth I was afraid that I was going to die.”
4. “During the childbirth I felt anxious/helpless/horrified.”

Four alternative answers follow each statement: “not at all,” “somehow,” “much,” and “very much.” Criterion A is fulfilled if either of the alternatives “much” or “very much” on item 1, 2 and/or 3, and 4 is marked(32, 33).

After the questions regarding criterion A, we have used the Posttraumatic Stress Disorder Checklist for DSM-5 (PCL-5) comprising the 20 PTSD symptoms (criterion B, C and D) to measure PTSD. The instrument contains 20 items, including three new PTSD symptoms (compared with the PTSD Checklist for DSM-V): blame, negative emotions and reckless or self-destructive behavior(34). A total-symptom score of zero to 80 can be obtained by summing the items. A score of 31-33 is optimal to determine PTSD symptoms and a score of ≥33 is recommended when further psychometric testing is not available(35, 36). Therefore, a score of ≥ 33 was considered to have symptoms of PTSD for this study. The instrument was used previously in Ethiopia(36).

Fear of child birth

The Wijma Delivery Expectation/Experience Questionnaire (W-DEQ) was used to measure fear of child birth. The W-DEQ has been designed specially to measure fear of child birth operationalized by the cognitive appraisal of the delivery. This 33-item rating scale has a 6-point

Likert scale as a response format, ranging from ' not at all' (= 0) to ' extremely' (= 5), yielding a score-range between 0 and 165. The Internal consistency and split-half reliability of the W-DEQ was checked in previous studies in Ethiopia with the Cronbach's alpha score of 0.932 (37, 38). A score of ≥ 85 was considered to have fear of child birth for this study(37, 38).

Social Support:

The Oslo 3-items social support scale with scores ranging from 3 to 14 was used to measure social support. The social support scores were categorized into poor or no social support for scores less than nine. Scores between 9 and 14 were considered moderate to strong support and merged together as "yes" for social support. The Oslo 3-items social support scale was validated and previously used in Ethiopia (39-41).

Data collection and quality control

Administering baseline questionnaire and diagnosis of direct and indirect maternal morbidities based on the WHO criteria, were done by health professionals working in the Gynecology and Obstetrics wards of the study Hospitals. The questionnaire consisted of a patient interview and record review. The interview was on socioeconomic status, medical and obstetric history and clinical symptoms. The record review was intended to extract information on selected laboratory tests and results for hemoglobin, HIV, malaria (RDT or smear) and glucometer (random blood sugar). The DASS-21 and PCL-5 were administered by health extension workers at the first, second and third home visit (6th, 12th and 18th week of postpartum period). Training was given for data collectors and supervision was done by the principal investigator. During the training process, data collectors carefully reviewed each question and conduct pretest before the study commences. The investigator and data collectors have checked the final version of the questionnaire and update as required based on the pretest.

Data processing and analysis

We used a three-wave, cross-lagged autoregressive structural equation modeling. The analysis was carried out by using Stata version 16 software. The Autoregressive Cross-lagged (ARCL) modeling strategy was used to examine the longitudinal relations between comorbidity of PTSD, depression, and anxiety. This modeling strategy incorporates three main components. First, the stability/autoregressive effects (effects of T1 comorbid depressive, anxiety and PTSD symptoms on their respective T2 variables). That means, later measures of a construct are predicted by earlier measures of the same construct. Second, the cross-lagged effects (effect of T1 depressive

symptoms on T2 comorbid PTSD and anxiety symptoms and of T1 comorbid PTSD and anxiety symptoms on T2 depressive symptoms). That means, earlier measures of depression predict later measures of comorbid PTSD and anxiety symptoms. This model can be extended to examine bi-directional relations such that earlier measures of PTSD predict later measures of comorbid depression and anxiety as well. Third, the synchronous associations between the unexplained variances of these variables at T1, T2 and T3(42, 43). We estimated the model fitness by using the comparative fit index (CFI), Tucker-Lewis Index (TLI) and the root-mean-square error of approximation (RMSEA). Both the TLI and CFI should be greater than 0.90, but the RMSEA value should be less than 0.08 to judge the model as reasonably fitting the data (13, 42). The aim of the analysis was to examine the cross-lagged effects of depressive, anxiety and PTSD symptoms, controlling for the confounder variables and stability/autoregressive effects. In addition, the direct and indirect relationships between the independent and dependent variables was also explored using the structural equation modeling. This allowed us to assess the strength of the hypothesized direct and indirect causal pathways. Estimated effects for which $p < 0.05$ were considered as being statistically significant.

Ethical considerations

This study was approved by the institutional review board of Bahir Dar Universit[illegible]ach study participant has given written informed consent before participating in the study. Assent was also obtained from teenage mothers whose age is less than 18 years, in addition to informed consent from their care givers. Using codes, passwords and limiting access to the data only for the investigators were the measures taken to ensure the confidentiality of the data. Data collectors read out and assisted participants to fill out the consent form if participants were unable to read and write

Results

Out of the 779 women recruited at baseline, 775(99.5%) of them participated at the first, second and third follow-up of the study (6th, 12th and 18th week of postpartum period). Four mothers were lost to follow up because of changing their place of living and going out of the study area. The mean age of the study participants was 26.3(4.36). Almost all of them 774(99.9%) were Amhara by ethnicity and 742(95.7%) were followers of Orthodox Christianity. Other socio-demographic characteristics of mothers are shown in Table 1.

Table 1: Socio-demographic characteristics of postpartum women in Northwest Ethiopia, 2021

Variables	Direct maternal morbidity		Total n (%)
	Yes. n (%)	No. n (%)	
**Age** [Mean(±SD) =26.33(±4.355)]			
**Residence**			
Urban	251 (32.4)	520 (67.1)	771(99.5)
Rural	1 (0.1)	3 (0.4)	4(0.5)
**Ethnicity**			
Amhara	252 (32.5)	522 (67.4)	774 (99.9)
Tigre	0 (0.0)	1 (0.1)	1 (0.1)
**Religion**			
Orthodox	241 (31.1)	501 (64.6)	742 (95.7)
Muslim	10 (1.3)	20 (2.6)	30 (3.9)
Protestant	1 (0.1)	2 (0.3)	3 (0.4)
**Education status**			
Illiterate/read and write	31 (4.0)	34 (4.4)	65 (8.4)
Grade 1-8	48 (6.2)	88 (11.4)	136 (17.5)
Grade 9-12	74 (9.5)	145 (18.7)	219 (28.3)
Certificate/Diploma	63 (8.1)	154 (19.9)	217 (28.0)
Degree and higher	36 (4.6)	102 (13.2)	138 (17.8)
**Occupation**			
Gov't employed	61 (7.9)	169 (21.8)	230 (29.7)
Merchant/Student	39 (5.0)	106 (13.7)	145 (18.7)
Housewife	141 (18.2)	226 (29.2)	367 (47.4)
Farmer/Daily laborer	11 (1.4)	22 (2.8)	33 (4.3)
**Marital Status**			
Married	246 (31.7)	516 (66.6)	762 (98.3)
Single/widowed/divorced	6 (0.8)	7 (0.9)	13 (1.7)
**Monthly expenditure**			
<= 3000 Ethiopian currency	48 (6.2)	158 (20.4)	206 (26.6)
3001-4000 Ethiopian currency	76 (9.8)	116 (15.0)	192 (24.8)
>= 4001 Ethiopian currency	128 (16.5)	249 (32.1)	377 (48.6)

Prevalence of Comorbid depression, anxiety and PTSD in the postpartum period

The prevalence of comorbid depression, anxiety and PTSD at the 6th, 12th and 18th week of postpartum period was computed and presented in Table 2. Comorbidity of anxiety and depression was the most common. PTSD demonstrated almost the same comorbidity with depression and with anxiety at all time points. The triple morbidity was most notable at the first follow up time.

Table 2: Prevalence of Comorbid depression, anxiety and PTSD among postpartum women in Northwest Ethiopia, 2021.

Postpartum Follow up time	Type of mental health disorder				Perceived traumatic childbirth n (%)
	PTSD and depression n (%)	PTSD and Anxiety n (%)	Anxiety and depression n (%)	Triple comorbidity n (%)	
**6th week**	73(9.4%)	72(9.3%)	112(14.5%)	72(9.3%)	306(39.5)
**12th week**	51(6.6%)	50(6.5%)	94(12.1%)	50(6.5%)	
**18th week**	23(3.0%)	23(3.0%)	63(8.1%)	23(3.0%)	
**Total number of women**	**775**	**775**	**775**	**775**	**775**

Stability and cross-lagged influence of depression, anxiety and PTSD on comorbidity of depression and anxiety

In order to assess the stability and cross-legged influence of depression, anxiety and PTSD on the co-morbidity of depression and anxiety, a cross-lagged autoregressive analysis was carried out and the result was presented in Figure 1 and Table 3. The model fits the data well with CFI = 0.97, TLI = 0.96 and RMSEA = 0.063.

Table 3: Results of a cross-lagged autoregressive analysis for the effect of depression, anxiety and PTSD on their comorbidities, among postpartum women in Northwest Ethiopia, 2021.

**The Autoregressive effect results**	**Standardized β (95%CI)**	**SE**	**P-value**
**Prediction of:**			
T2 Depression & PTSD comorbidity by T1 comorbidity	0.70(0.63, 0.76)	0.03	<0.001
T3 Depression & PTSD comorbidity by T2 comorbidity	0.44(0.37, 0.50)	0.03	<0.001
T2 Anxiety & PTSD comorbidity by T1 comorbidity	0.70(0.63, 0.76)	0.03	<0.001
T3 Anxiety & PTSD comorbidity by T2 comorbidity	0.45(0.39, 0.51)	0.03	<0.001
T2 Depression & anxiety comorbidity by T1 comorbidity	0.76(0.71, 0.80)	0.02	<0.001
T3 Depression & anxiety comorbidity by T2 comorbidity	0.72(0.68, 0.76)	0.02	<0.001
T2 Multimorbidity by T1 Multimorbidity	0.68(0.61, 0.74)	0.03	<0.001
T3 Multimorbidity by T2 Multimorbidity	0.45(0.39, 0.51)	0.03	<0.001
**The Cross-lagged effect results**	**Standardized β (95%CI)**	**SE**	**P-value**
**Prediction of:**			
T2 Depression & anxiety comorbidity by T1 anxiety	0.15(0.11, 0.20)	0.02	<0.001
T3 Depression & anxiety comorbidity by T2 anxiety	0.08(0.03, 0.13)	0.02	0.001
T2 Anxiety & PTSD comorbidity by T1 PTSD	0.12(0.05, 0.19)	0.04	<0.001
T3 Anxiety & PTSD comorbidity by T2 anxiety	0.25(0.18, 0.31)	0.03	<0.001
T2 Depression & PTSD comorbidity by T1 PTSD	0.13(0.06, 0.20)	0.04	<0.001
T3 Depression & PTSD comorbidity by T2 anxiety	0.26(0.19, 0.32)	0.03	<0.001
T2 Multimorbidity by T1 PTSD	0.15(0.07, 0.22)	0.04	<0.001
T3 Multimorbidity by T2 anxiety	0.25(0.18, 0.31)	0.03	<0.001

As can be seen in Fig. 1 and Table 3, the stability of suffering from comorbidity of depression and anxiety was high: participants suffering from comorbidity of depression and anxiety in Time 1 continue to suffer from this comorbidity over time. Time 1 comorbid anxiety and depression was associated with that of Time 2, and this pattern was extended to Time 3. More importantly, the analyses revealed that anxiety symptoms predicted co-morbidity between depression and anxiety. In other words, suffering from anxiety symptoms increased the likelihood of co-morbidity of depression and anxiety in subsequent waves of measurement. However, suffering from depression

or PTSD symptoms did not change the likelihood of co-morbidity between depression and anxiety symptoms in subsequent waves of measurement.

Stability and cross-lagged influence of depression, anxiety and PTSD on comorbidity of depression and PTSD

The result of another cross-lagged autoregressive analysis for the stability and cross-legged influence of depression, anxiety and PTSD on the co-morbidity of depression and PTSD, was indicated in Figure 2 and Table 3. This model also fits the data well with CFI = 0.98, TLI = 0.96 and RMSEA = 0.062. As can be seen in Fig. 2 and Table 3, the stability of suffering from comorbidity of depression and PTSD was high: participants suffering from co-morbidity of depression and PTSD in Time 1 tended to suffer from this co-morbidity over time. The analysis also revealed that Time 1 PTSD predicted comorbidity of depression and PTSD in Time 2. Similarly, Time 2 anxiety predicted comorbidity of depression and PTSD in Time 3. However, as can be seen in Table 3, the association between Time 2 anxiety and comorbidity of depression and PTSD ($\beta = 0.26$, p-value <0.0001), was stronger than the association between Time 1 PTSD and comorbidity of depression and PTSD ($\beta = 0.13$, p-value =0.0003).

Stability and cross-lagged influence of depression, anxiety and PTSD on comorbidity of anxiety and PTSD

To examine the stability and cross-legged influence of depression, anxiety and PTSD on the co-morbidity of anxiety and PTSD, another cross-lagged autoregressive analysis was carried out and the result was presented in Figure 3 and Table 3. This model also fits the data well with CFI = 0.98, TLI = 0.96 and RMSEA = 0.063. The autoregressive analyses revealed that, comorbidity of anxiety and PTSD symptoms were stable over time, which indicates that participants who suffered from comorbidity of anxiety and PTSD in Time 1, tended to suffer from this comorbidity both in Time 2 and Time 3. Moreover, comorbidity of anxiety and PTSD in Time 2, was predicted by PTSD in Time 1 and this comorbidity in Time 3 was predicted by anxiety in Time 2. However, the association between Time 2 anxiety and comorbidity of anxiety and PTSD ($\beta = 0.25$, p-value <0.0001), was stronger than the association between Time 1 PTSD and comorbidity of anxiety and PTSD ($\beta = 0.12$, p-value =0.0005).

Stability and cross-lagged influence of depression, anxiety and PTSD on triple comorbidity

We have also carried out a cross-lagged autoregressive analysis to examine whether anxiety, depression and PTSD predict triple comorbidity and the result was presented in Figure 4 and Table 3. The model fits the data well with CFI = 0.97, TLI = 0.96 and RMSEA = 0.07.

The autoregressive analyses indicated that triple comorbidity of anxiety, depression and PTSD symptoms were stable over time which indicates that participants who suffered from triple comorbidity in Time 1 tended to suffer from full comorbidity both in Time 2 and Time 3. The cross-lagged effect revealed that Time 1 PTSD predicted triple comorbidity in Time 2 and Time 2 anxiety predicted triple comorbidity in Time 2. However, the association of Time 2 anxiety with triple comorbidity in Time 3 ($\beta = 0.25$, p-value <0.0001), was stronger than the association of Time 1 PTSD with Time 2 triple comorbidity ($\beta = 0.15$, p-value =0.00006).

Over all, anxiety predicted comorbidity of depression with anxiety at the second and third follow up period, depression with PTSD, anxiety with PTSD and triple comorbidity at the third follow up period stronger than depression and PTSD.

Direct, indirect and total effects of variables on comorbidity of anxiety and PTSD

As shown in Figure 2 and Table 4, fear of childbirth and gravidity had a direct positive association on Time 1 anxiety and PTSD comorbidity and an indirect positive association with these comorbidities on Time 2 and Time 3. Direct maternal morbidity had a direct positive association with comorbidity of Time 1 anxiety and PTSD score. It had also a direct and an indirect positive effect on comorbidity of Time 2 anxiety and PTSD and an indirect effect on Time 3 comorbidity.

Time 1 anxiety, Time 1 PTSD and comorbidity of Time 1 anxiety with PTSD had a positive indirect association on Time 3 comorbidity of anxiety and PTSD. Parity and family size had a direct negative effect on Time 1 anxiety and PTSD comorbidity and an indirect negative effect on Time 2 and Time 3 comorbidities (see Fig. 2 and Table 4).

Direct, indirect and total effects of variables on comorbidity of depression and PTSD

There was a direct positive association between direct maternal morbidity and comorbidity of Time 1 depression and PTSD score. It had also a direct and an indirect positive effect on comorbidity of Time 2 depression and PTSD and an indirect effect on Time 3 comorbidity. Parity and family size had a direct negative effect on Time 1 depression and PTSD comorbidity and an indirect negative effect on Time 2 and Time 3 comorbidities (see Fig. 3 and Table 4). In contrast, fear of childbirth and gravidity had a direct positive association on Time 1 depression and PTSD comorbidity and an indirect positive association on Time 2 and Time 3 comorbidities.

Table 4: Direct, Indirect and total effects of variables associated with comorbidities of depression, anxiety and PTSD, among postpartum women in Northwest Ethiopia, 2021.

Variable's pathway	Type of mental health disorder at each follow up time								
	T1 Anxiety & PTSD			T2 Anxiety & PTSD			T3 Anxiety & PTSD		
	Direct effect	Indirect effect	Total effect	Direct effect	Indirect effect	Total effect	Direct effect	Indirect effect	Total effect
	β (SE)	β (SE)	β (SE)	β (SE)	β (SE)	β (SE)	β (SE)	β (SE)	β (SE)
Direct morbidity Yes	0.19(0.03) [a]	No path	0.19(0.03) [a]	No path	0.15(0.01) [a]	0.15(0.01) [a]	No path	0.11(0.01) [a]	0.11(0.01) [a]
Fear of childbirth Yes	0.23(0.03) [a]	No path	0.23(0.03) [a]	No path	0.20(0.0003) [a]	0.20(0.0003) [a]	No path	0.15(0.0001) [a]	0.15(0.0001) [a]
Gravidity	0.24(0.07) [a]	No path	0.24(0.07) [a]	No path	0.20(0.01) [a]	0.20(0.01) [a]	No path	0.15(0.01) [a]	0.15(0.01) [a]
Parity	-0.16(0.08) [c]	No path	-0.16(0.08) [c]	No path	-0.13(0.01) [c]	-0.13(0.01) [c]	No path	-0.12(0.01) [b]	-0.12(0.01) [b]
Family size	-0.09(0.05) [c]	No path	-0.09(0.05) [c]	No path	-0.08(0.01) [c]	-0.08(0.01) [c]	No path	-0.03(0.002) [c]	-0.03(0.002) [c]
Traumatic birth Yes	0.31(0.03) [a]	No path	0.31(0.03) [a]	No path	0.26(0.01) [a]	0.26(0.01) [a]	No path	0.18(0.01) [a]	0.18(0.01) [a]
Indirect morbidity Yes	No path	0.13(0.02) [a]	0.13(0.02) [a]	No path	0.10(0.01) [a]	0.10(0.01) [a]	No path	0.08(0.005) [a]	0.08(0.005) [a]
T1 PTSD							No path	0.06(0.0002) [a]	0.06(0.0002) [a]
T1 anxiety							No path	0.23(0.001) [a]	0.23(0.001) [a]
T1 anxiety & PTSD							No path	0.31(0.02) [a]	0.31(0.02) [a]

Variable's pathway	Type of mental health disorder at each follow up time								
	T1 Depression & PTSD			T2 Depression & PTSD			T3 Depression & PTSD		
	Direct effect	Indirect effect	Total effect	Direct effect	Indirect effect	Total effect	Direct effect	Indirect effect	Total effect
	β (SE)	β (SE)	β (SE)	β (SE)	β (SE)	β (SE)	β (SE)	β (SE)	β (SE)
Direct morbidity Yes	0.19(0.03) [a]	No path	0.19(0.03) [a]	No path	0.15(0.01) [a]	0.15(0.01) [a]	No path	0.11(0.01) [a]	0.11(0.01) [a]
Fear of childbirth Yes	0.23(0.03) [a]	No path	0.23(0.03) [a]	No path	0.20(0.0003) [a]	0.20(0.0003) [a]	No path	0.15(0.0001) [a]	0.15(0.0001) [a]
Gravidity	0.25(0.07) [a]	No path	0.25(0.07) [a]	No path	0.21(0.01) [a]	0.21(0.01) [a]	No path	0.15(0.01) [a]	0.15(0.01) [a]
Parity	-0.17(0.08) [c]	No path	-0.17(0.08) [c]	No path	-0.15(0.01) [c]	-0.15(0.01) [c]	No path	-0.12(0.01) [b]	-0.12(0.01) [b]
Family size	-0.09(0.05) [c]	No path	-0.09(0.05) [c]	No path	-0.08(0.01) [c]	-0.08(0.01) [c]		-0.03(0.002) [c]	-0.03(0.002) [c]
Traumatic birth Yes	0.32(0.03) [a]	No path	0.32(0.03) [a]	No path	0.26(0.01) [a]	0.26(0.01) [a]	No path	0.18(0.01) [a]	0.18(0.01) [a]
Indirect morbidity Yes	No path	0.13(0.02) [a]	0.13(0.02) [a]	No path	0.10(0.01) [a]	0.10(0.01) [a]	No path	0.08(0.005) [a]	0.08(0.005) [a]
T1 PTSD							No path	0.06(0.0002) [a]	0.06(0.0002) [a]
T1 anxiety							No path	0.24(0.001) [a]	0.24(0.001) [a]
T1 Dep. & PTSD							No path	0.30(0.02) [a]	0.30(0.02) [a]

[a] p-value ≤0.001, [b] p-value <0.01, [c] p-value <0.05, β is standard estimate

Direct, indirect and total effects of variables on multimorbidity and comorbidity of depression and anxiety

As can be seen in Fig. 1 and Table 5, direct maternal morbidity had a direct positive effect on Time 1 comorbidity of anxiety and depression. It had also a direct and an indirect positive effect on Time 2 anxiety and depression comorbidity and an indirect effect on Time 3 comorbidity. Time 1 anxiety and comorbidity of Time 1 anxiety and depression had also a positive indirect effect on Time 3 comorbidity of anxiety and depression (see Fig. 1 and Table 5).

With regard to triple comorbidity, direct maternal morbidity, fear of childbirth, gravidity and perceived traumatic childbirth were found to have a direct positive association with it on Time 1. Parity had a direct negative effect on Time 1 triple comorbidity and an indirect negative effect on Time 2 and Time 3 comorbidities (see Fig. 4 and Table 5). In contrast, indirect maternal morbidity was found to have an indirect positive association with triple comorbidity at all time points. Time 1 anxiety, Time 1 PTSD and Time 1 triple morbidity had also a positive indirect association on Time 3 triple comorbidity.

Table 5: Direct, Indirect and total effects of variables associated with multimorbidity and comorbidity of depression and anxiety, among postpartum women in Northwest Ethiopia, 2021.

Variable's pathway	Type of mental health disorder at each follow up time								
	T1 Anxiety & depression			T2 Anxiety & depression			T3 Anxiety & depression		
	Direct effect	Indirect	Total effect	Direct effect	Indirect	Total effect	Direct effect	Indirect	Total effect
	β (SE)	β (SE)	β (SE)	β (SE)	β (SE)	β (SE)	β (SE)	β (SE)	β (SE)
Direct morbidity									
Yes	0.26(0.03) [a]	No path	0.26(0.03) [a]	0.09(0.02) [a]	0.23(0.02) [a]	0.32(0.02) [a]	No path	0.25(0.02) [a]	0.25(0.02) [a]
Fear of childbirth									
Yes	0.28(0.03) [a]	No path	0.28(0.03) [a]	No path	0.24(0.0004) [a]	0.24(0.0004) [a]	No path	0.19(0.0002) [a]	0.19(0.0002) [a]
Gravidity	0.21(0.07) [b]	No path	0.21(0.07) [b]	No path	0.19(0.02) [b]	0.19(0.02) [b]	No path	0.15(0.01) [b]	0.15(0.01) [b]
Parity	-0.23(0.07) [a]	No path	-0.23(0.07) [a]	No path	-0.21(0.02) [a]	-0.21(0.02) [a]	No path	-0.16(0.01) [a]	-0.16(0.01) [a]
Social support	-0.15(0.04) [a]	No path	-0.15(0.04) [a]	No path	-0.15(0.01) [a]	-0.15(0.01) [a]	No path	-0.12(0.003) [a]	-0.12(0.003) [a]
Traumatic birth									
Yes	0.23(0.03) [a]	No path	0.23(0.03) [a]	No path	0.21(0.02) [a]	0.21(0.02) [a]	No path	0.17(0.01) [a]	0.17(0.01) [a]
Indirect morbidity									
Yes	No path	0.18(0.02) [a]	0.18(0.02) [a]	No path	0.22(0.02) [a]	0.22(0.02) [a]	No path	0.17(0.01) [a]	0.17(0.01) [a]
T1 Anxiety							No path	0.19(0.01) [a]	0.19(0.01) [a]
T1 Dep. & anxiety							No path	0.54(0.02) [a]	0.54(0.02) [a]
**Variable's pathway**	**Type of mental health disorder at each follow up time**								
	**T1 Multimorbidity**			**T2 Multimorbidity**			**T3 Multimorbidity**		
	Direct effect	Direct effect	Direct effect	Direct effect	Direct effect	Direct effect	Direct effect	Direct effect	Direct effect
	β (SE)	β (SE)	β (SE)	β (SE)	β (SE)	β (SE)	β (SE)	β (SE)	β (SE)
Direct morbidity									
[illegible]	[illegible]	[illegible]	[illegible]	[illegible]	[illegible]	[illegible]	[illegible]	[illegible]	[illegible]
Fear of childbirth									
Yes	0.23(0.03) [a]	No path	0.23(0.03) [a]	No path	0.20(0.0003) [a]	0.20(0.0003) [a]	No path	0.15(0.0001) [a]	0.15(0.0001) [a]
Gravidity	0.22(0.07) [b]	No path	0.22(0.07) [b]	No path	0.18(0.01) [b]	0.18(0.01) [b]	No path	0.14(0.01) [a]	0.14(0.01) [a]
Parity	-0.22(0.07) [b]	No path	-0.22(0.07) [b]	No path	-0.19(0.01) [b]	-0.19(0.01) [b]	No path	-0.14(0.01) [a]	-0.14(0.01) [a]
Traumatic birth									
Yes	0.31(0.03) [a]	No path	0.31(0.03) [a]	No path	0.25(0.01) [a]	0.25(0.01) [a]	No path	0.17(0.01) [a]	0.17(0.01) [a]
Indirect morbidity									
Yes	No path	0.14(0.02) [a]	0.14(0.02) [a]	No path	0.10(0.01) [a]	0.10(0.01) [a]	No path	0.06(0.005) [a]	0.06(0.005) [a]
T1 PTSD							No path	0.07(0.0002) [a]	0.07(0.0002) [a]
T1 Anxiety							No path	0.23(0.001) [a]	0.23(0.001) [a]
T1 multimorbidity							No path	0.30(0.01) [a]	0.30(0.01) [a]

[a] p-value ≤0.001, [b] p-value <0.01, β is standard estimate

Discussion

Comorbidity of perinatal depression, anxiety and PTSD

Comorbidity of depression and anxiety was more frequently observed in our study (14.5%) and is consistent with previous literatures(26, 27). The coexistence of PTSD with depression (9.4%) and PTSD with anxiety (9.3%) was equally prevalent after childbirth which is ~~in~~ congruent with a previous study (27). This suggests that women with depression or anxiety are more prone to experience PTSD symptoms which was confirmed by the cross-lagged autoregressive result of this study. The rates of multimorbidity after childbirth were 9.3% ,6.5% and 3.0% at the 6th, 12th and

18th week of postpartum which is consistent with the finding of previous literatures(27, 44). The existence of comorbidities suggests that when a mother is suspected in any of depressive, anxiety or PTSD symptoms, screening for the other disorder symptoms is important for treatment purposes.

Cross-lagged autoregressive effects of depression, anxiety and PTSD on their comorbidities

Measuring the relation patterns of depression, anxiety and PTSD with their comorbidities over time revealed that anxiety is a predictor of the comorbidity between depression and anxiety throughout the follow up period. It also predicts the triple comorbidity, comorbidity of depression with PTSD and comorbidity of anxiety with PTSD from the second to the third follow up period. This implies that, anxiety is a causal risk factor for both depression and PTSD in the postpartum period which is consistent with previous studies that anxiety was found to be risk factor for depression and PTSD among childbearing women(13, 23, 45-48).

Direct, indirect and total effects of variables associated with comorbidity of depression, anxiety and PTSD

In this study, direct maternal morbidity has a direct and positive association with comorbidity of anxiety with depression at the first and second follow up period. It also increases the risk of comorbidity between anxiety and PTSD, depression and PTSD and triple comorbidity with a direct positive association at the first follow up period. It has also an indirect positive association with these events at the second and third follow up period through their respective previous waves of measurement. Indirect maternal morbidity was also found to have a positive indirect association with all the above events through direct maternal morbidity and their respective previous waves of measurement. Previous literatures have reported similar findings that the onset of depression, post-traumatic stress and anxiety are higher among women whose pregnancy included obstetrical complications, compared with women with uneventful pregnancies(49-53).

Similar with previous literatures(18, 50, 54, 55), fear of childbirth, gravidity and perceived traumatic childbirth were found to increase the risk of comorbid depression, anxiety and PTSD directly at the first follow up period and indirectly at the second and third follow up period. In contrast, multiparity decrease the risk of comorbid depression, anxiety and PTSD directly at the first follow up period and indirectly at the second and third follow up period. Higher family siz[illegible] was found to be protective of comorbid depression with PTSD and anxiety with PTSD directly at the first follow up period and indirectly at the second and third follow up period.

Higher social support also decreases the risk of comorbidity between depression, anxiety and PTSD directly at the first follow up period and indirectly at the second and third follow up period which is in congruent with previous literatures(1, 2). This might be due to the buffering effect of higher social support on negative cognitions since it provides needed social, emotional and physical provisions(1).

Strength and Limitation of the study

Strength of this study is the investigation of longitudinal associations of depression, anxiety and PTSD with their comorbidities using a cross-lagged autoregressive structural equation modeling. However, this study was not without limitations. First, antenatal factors like depression and anxiety during pregnancy and prior PTSD which may influence the comorbidity rates of these events in the postpartum period were not included in the study. Thus, it would have been better to include antenatal depression, anxiety and prior PTSD as a confounding variable. Therefore, further studies are required to clarify this issue. Second, self-report questionnaires rather than clinical interviews were used to assess anxiety, depression and PTSD which might inflate the comorbid prevalence rates. Although spurious findings were sometimes the limitation of cross-lagged structural equation modeling, we have collected longitudinal data on anxiety, depression and PTSD at the 6th, 12th and 18th week of postpartum period to determine the temporal sequencing of these disorders. Therefore, the issue of spurious findings during the evaluation of whether depression or anxiety or PTSD preceded comorbidities of these disorders or vice versa was controlled and minimized by doing so.

Conclusion and Recommendation

The findings of this study showed that comorbidity of anxiety with depression was the most common among the events of comorbidity between anxiety, depression and PTSD at the 6th, 12th and 18th week of postpartum period. In addition, the coexistence of PTSD with depression, PTSD with anxiety and triple comorbidity was equally prevalent after childbirth throughout the follow up period.

Furthermore, investigation of the chronological relations of depression, anxiety and PTSD on their comorbidities indicated that anxiety was a causal risk factor for comorbidity of anxiety with depression, comorbidity of depression with PTSD and for triple comorbidity stronger than depression and PTSD. In the current study, direct maternal morbidity, fear of childbirth, higher gravidity, perceived traumatic childbirth and indirect maternal morbidity were found to have a

direct and indirect positive association with comorbidities of depression, anxiety and PTSD. In addition, higher parity, higher family size and higher social support have a direct and indirect negative association.

Therefore, there should be effective postnatal screening to identify women with psychological problems. Early detection and treatment of anxiety may reduce the likelihood of postnatal comorbid psychological disorders. Women with anxiety symptoms should also be screened for postpartum depression and PTSD symptoms.

Women with direct and indirect maternal morbidities should be identified and treated early, in order to reduce the subsequent burden of comorbid anxiety, depression and PTSD. Adequate information about birth procedures and response to their needs should be given for women with fear of childbirth and perceived traumatic birth to decrease negative emotions of women and fear of childbirth. This would prevent the subsequent comorbid anxiety, depressive and PTSD symptoms. Interventions targeting to encourage social support may also help in increasing mothers' coping and buffer negative cognitions so as to prevent symptoms of comorbid anxiety, depression and PTSD.

Author affiliations

[1*]Department of Public Health, College of Health Sciences, Debre Tabor University, Debre Tabor, Ethiopia

[1,2] School of Public Health, College of Medicine and Health Sciences, Bahir Dar University, Bahir Dar, Ethiopia

[3] School of Public Health, College of Medicine and Health Sciences, University of Gondar

Acknowledgements: The authors would like to acknowledge the heads of Debre Tabor Hospital, Addis Zemen Hospital, Estie Hospital and Nefas Mewcha Hospital for their cooperation on the data collection of this study. The authors are also grateful to the study participants for their dedicated time and volunteer participation.

Authors' contributions: MT was investigator, involved in proposal writing, designing, and recruitment and training of supervisors and data collectors, analysis and write-up and in all stages of the project implementation. He did most of the analysis and write up of the paper. GD and TA contributed in the designing of the methodology, lead investigator and involved in designing of project proposal, design of questionnaires, supervision and involved in giving comments in the

progress of the project and final approval of the paper. All authors read and approved the final manuscript.

Funding: This study was conducted as part of PhD degree fulfillment supported by Bahir Dar University and Debre Tabor University.

Competing interests: None declared.

Ethics approval: Ethical approval was obtained from Institutional Review Board of Bahir Dar University (Reference number: 00225/2020).

Data availability statement: Extra data is available from the corresponding author upon reasonable request.

References:

1. Oh W, Muzik M, McGinnis EW, Hamilton L, Menke RA, Rosenblum KL. Comorbid trajectories of postpartum depression and PTSD among mothers with childhood trauma history: Course, predictors, processes and child adjustment. Journal of affective disorders. 2016;200:133-41.

2. van der Zee-van AI, Boere-Boonekamp MM, Groothuis-Oudshoorn CG, Reijneveld SA. Postpartum depression and anxiety: a community-based study on risk factors before, during and after pregnancy. Journal of affective disorders. 2021;286:158-65.

3. Association AP. Diagnostic and statistical manual of mental disorders (DSM-5®): American Psychiatric Pub; 2013.

4. Gelaye B, Rondon MB, Araya R, Williams MA. Epidemiology of maternal depression, risk factors, and child outcomes in low-income and middle-income countries. The Lancet Psychiatry. 2016;3(10):973-82.

5. Organization WH. Maternal mental health and child health and development in resource-constrained settings: report of a UNFPA. World Health Organization; 2009.

6. Sawyer A, Ayers S, Smith H. Pre-and postnatal psychological wellbeing in Africa: a systematic review. Journal of affective disorders. 2010;123(1-3):17-29.

7. Hartley M, Tomlinson M, Greco E, Comulada WS, Stewart J, Le Roux I, et al. Depressed mood in pregnancy: prevalence and correlates in two Cape Town peri-urban settlements. Reproductive health. 2011;8(1):1-7.

8. Kakyo TA, Muliira JK, Mbalinda SN, Kizza IB, Muliira RS. Factors associated with depressive symptoms among postpartum mothers in a rural district in Uganda. Midwifery. 2012;28(3):374-9.

9. Fairbrother N, Janssen P, Antony MM, Tucker E, Young AH. Perinatal anxiety disorder prevalence and incidence. Journal of affective disorders. 2016;200:148-55.
10. Dennis CL, Falah‐ Hassani K, Brown H, Vigod S. Identifying women at risk for postpartum anxiety: a prospective population‐ based study. Acta Psychiatrica Scandinavica. 2016;134(6):485-93.
11. Dikmen-Yildiz P, Ayers S, Phillips L. Longitudinal trajectories of post-traumatic stress disorder (PTSD) after birth and associated risk factors. Journal of affective disorders. 2018;229:377-85.
12. Geller PA, Stasko EC. Effect of previous posttraumatic stress in the perinatal period. Journal of Obstetric, Gynecologic & Neonatal Nursing. 2017;46(6):912-22.
13. Haagen JF, Moerbeek M, Olde E, Van Der Hart O, Kleber RJ. PTSD after childbirth: A predictive ethological model for symptom development. Journal of affective disorders. 2015;185:135-43.
14. Anderson CA. The trauma of birth. Health care for women international. 2017;38(10):999-1010.
15. de Graaff LF, Honig A, van Pampus MG, Stramrood CA. Preventing post‐ traumatic stress disorder following childbirth and traumatic birth experiences: a systematic review. Acta obstetricia et gynecologica Scandinavica. 2018;97(6):648-56.
16. Alcorn KL, O'Donovan A, Patrick JC, Creedy D, Devilly GJ. A prospective longitudinal study of the prevalence of post-traumatic stress disorder resulting from childbirth events. Psychological medicine. 2010;40(11):1849-59.
17. Grekin R, O'Hara MW. Prevalence and risk factors of postpartum posttraumatic stress disorder: a meta-analysis. Clinical psychology review. 2014;34(5):389-401.
18. Ayers S, Bond R, Bertullies S, Wijma K. The aetiology of post-traumatic stress following childbirth: a meta-analysis and theoretical framework. Psychological medicine. 2016;46(6):1121-34.
19. Dekel S, Stuebe C, Dishy G. Childbirth Induced Posttraumatic Stress Syndrome: A Systematic Review of Prevalence and Risk Factors. Front Psychol. 2017;8:560.
20. Yildiz PD, Ayers S, Phillips L. The prevalence of posttraumatic stress disorder in pregnancy and after birth: A systematic review and meta-analysis. Journal of affective disorders. 2017;208:634-45.

21. Garthus-Niegel S, von Soest T, Vollrath ME, Eberhard-Gran M. The impact of subjective birth experiences on post-traumatic stress symptoms: a longitudinal study. Archives of women's mental health. 2013;16(1):1-10.
22. Modarres M, Afrasiabi S, Rahnama P, Montazeri A. Prevalence and risk factors of childbirth-related post-traumatic stress symptoms. BMC pregnancy and childbirth. 2012;12(1):1-6.
23. Andersen LB, Melvaer LB, Videbech P, Lamont RF, Joergensen JS. Risk factors for developing post- traumatic stress disorder following childbirth: a systematic review. Acta obstetricia et gynecologica Scandinavica. 2012;91(11):1261-72.
24. Harrison S, Ayers S, Quigley M, Stein A, Alderdice F. Prevalence and factors associated with postpartum posttraumatic stress in a population-based maternity survey in England. Journal of affective disorders. 2021;279:749-56.
25. Sommerlad S, Schermelleh-Engel K, La Rosa VL, Louwen F, Oddo-Sommerfeld S. Trait anxiety and unplanned delivery mode enhance the risk for childbirth-related post-traumatic stress disorder symptoms in women with and without risk of preterm birth: A multi sample path analysis. PloS one. 2021;16(8):e0256681.
26. Falah-Hassani K, Shiri R, Dennis C-L. Prevalence and risk factors for comorbid postpartum depressive symptomatology and anxiety. Journal of affective disorders. 2016;198:142-7.
27. Dikmen-Yildiz P, Ayers S, Phillips L. Depression, anxiety, PTSD and comorbidity in perinatal women in Turkey: A longitudinal population-based study. Midwifery. 2017;55:29-37.
28. Agius A, Xuereb RB, Carrick-Sen D, Sultana R, Rankin J. The co-existence of depression, anxiety and post-traumatic stress symptoms in the perinatal period: a systematic review. Midwifery. 2016;36:70-9.
29. Barreix M, Barbour K, McCaw-Binns A, Chou D, Petzold M, Gichuhi GN, et al. Standardizing the measurement of maternal morbidity: Pilot study results. International journal of gynaecology and obstetrics: the official organ of the International Federation of Gynaecology and Obstetrics. 2018;141 Suppl 1:10-9.
30. Yeshaw Y, Mossie A. Depression, anxiety, stress, and their associated factors among Jimma University staff, Jimma, Southwest Ethiopia, 2016: a cross-sectional study. Neuropsychiatr Dis Treat. 2017;13:2803-12.

31. Abebe AM, Kebede YG, Mengistu F. Prevalence of Stress and Associated Factors among Regular Students at Debre Birhan Governmental and Nongovernmental Health Science Colleges North Showa Zone, Amhara Region, Ethiopia 2016. Psychiatry J. 2018;2018:7534937.
32. Wijma K, Söderquist J, Wijma B. Posttraumatic stress disorder after childbirth: a cross sectional study. Journal of anxiety disorders. 1997;11(6):587-97.
33. King L, McKenzie-McHarg K, Horsch A. Testing a cognitive model to predict posttraumatic stress disorder following childbirth. BMC pregnancy and childbirth. 2017;17(1):1-12.
34. Weathers FW, Litz, B.T., Keane, T.M., Palmieri, P.A., Marx, B.P., & Schnurr, P.P. The PTSD Checklist for DSM-5 (PCL-5). Scale available from the National Center for PTSD at www.ptsd.va.gov. 2013.
35. Verhey R, Chibanda D, Gibson L, Brakarsh J, Seedat S. Validation of the posttraumatic stress disorder checklist - 5 (PCL-5) in a primary care population with high HIV prevalence in Zimbabwe. BMC psychiatry. 2018;18(1):109-.
36. Smyth-Dent K, Fitzgerald J, Hagos Y. A Field Study on the EMDR Integrative Group Treatment Protocol for Ongoing Traumatic Stress Provided to Adolescent Eritrean Refugees Living in Ethiopia. Psychology and Behavioral Science International Journal. 2019;12(4):1-12.
37. Gelaw T, Ketema TG, Beyene K, Gurara MK, Ukke GG. Fear of childbirth among pregnant women attending antenatal care in Arba Minch town, southern Ethiopia: a cross-sectional study. BMC pregnancy and childbirth. 2020;20(1):1-7.
38. Yetwale A, Melkamu E. Fear of Childbirth and Associated Factors Among Pregnant Mothers Who Attend Antenatal Care Service at Jinka Public Health Facilities, Jinka Town, Southern Ethiopia. International Journal of Childbirth. 2021.
39. Bezabh YH, Abebe SM, Fanta T, Tadese A, Tulu M. Prevalence and associated factors of post-traumatic stress disorder among emergency responders of Addis Ababa Fire and Emergency Control and Prevention Service Authority, Ethiopia: institution-based, cross-sectional study. BMJ open. 2018;8(7):e020705.
40. Asnakew S, Shumet S, Ginbare W, Legas G, Haile K. Prevalence of post-traumatic stress disorder and associated factors among Koshe landslide survivors, Addis Ababa, Ethiopia: a community-based, cross-sectional study. BMJ open. 2019;9(6):e028550.

41. Denur M, Tesfaw G, Yohannis Z. The magnitude and correlates of common mental disorder among outpatient medical patients in Ethiopia: an institution based cross-sectional study. BMC research notes. 2019;12(1):360.
42. Ginzburg K, Ein-Dor T, Solomon Z. Comorbidity of posttraumatic stress disorder, anxiety and depression: a 20-year longitudinal study of war veterans. Journal of affective disorders. 2010;123(1-3):249-57.
43. Shahar G, Herishanu-Gilutz S, Holcberg G, Kofman O. In first-time mothers, post-partum depressive symptom prospectively predict symptoms of post-traumatic stress. Journal of affective disorders. 2015;186:168-70.
44. Agius A, Xuereb RB, Carrick-Sen D, Sultana R, Rankin J. The co-existence of depression, anxiety and post-traumatic stress symptoms in the perinatal period: A systematic review. Midwifery. 2016;36:70-9.
45. Norhayati M, Hazlina NN, Asrenee A, Emilin WW. Magnitude and risk factors for postpartum symptoms: a literature review. Journal of affective disorders. 2015;175:34-52.
46. Peñacoba-Puente C, Marín-Morales D, Carmona-Monge FJ, Velasco Furlong L. Post-partum depression, personality, and cognitive-emotional factors: A longitudinal study on Spanish pregnant women. Health care for women international. 2016;37(1):97-117.
47. Alipour Z, Lamyian M, Hajizadeh E. Anxiety and fear of childbirth as predictors of postnatal depression in nulliparous women. Women and Birth. 2012;25(3):e37-e43.
48. Zaers S, Waschke M, Ehlert U. Depressive symptoms and symptoms of post-traumatic stress disorder in women after childbirth. Journal of psychosomatic obstetrics and gynaecology. 2008;29(1):61-71.
49. Pranal M, Legrand A, de Chazeron I, Llorca P-M, Vendittelli F. Prevalence of maternal psychological disorders after immediate postpartum haemorrhage: a repeated cross-sectional study-the PSYCHE* study protoco. BMJ open. 2019;9(9):e027390.
50. Grekin R, O’Hara MW, Brock RL. A model of risk for perinatal posttraumatic stress symptoms. Archives of women's mental health. 2021;24(2):259-70.
51. Dadi AF, Mwanri L, Woodman RJ, Azale T, Miller ER. Causal mechanisms of postnatal depression among women in Gondar town, Ethiopia: application of a stress-process model with generalized structural equation modeling. Reproductive health. 2020;17(1):1-15.

52. Furuta M, Sandall J, Bick D. A systematic review of the relationship between severe maternal morbidity and post-traumatic stress disorder. BMC pregnancy and childbirth. 2012;12:125.
53. Dikmen-Yildiz P, Ayers S, Phillips L. Factors associated with post-traumatic stress symptoms (PTSS) 4–6 weeks and 6 months after birth: A longitudinal population-based study. Journal of affective disorders. 2017;221:238-45.
54. Gokce IsbIr G, Inc IF, Bektas M, Dikmen Yildiz P, Ayers S. Risk factors associated with post-traumatic stress symptoms following childbirth in Turkey. Midwifery. 2016;41:96-103.
55. Andersen LB, Melvaer LB, Videbech P, Lamont RF, Joergensen JS. Risk factors for developing post-traumatic stress disorder following childbirth: a systematic review. Acta obstetricia et gynecologica Scandinavica. 2012;91(11):1261-72.

Legend:

Figure 1: Longitudinal stability and cross-lagged effects of depression, anxiety and PTSD on the comorbidity of depression and anxiety. ***Note:*** All β's are standardized estimates, only significant paths are included in the path diagram.

Figure 2: Longitudinal stability and cross-lagged effects of depression, anxiety and PTSD on the comorbidity of depression and PTSD. ***Note:*** All β's are standardized estimates, only significant paths are included in the path diagram.

Figure 3: Longitudinal stability and cross-lagged effects of depression, anxiety and PTSD on the comorbidity of anxiety and PTSD. ***Note:*** All β's are standardized estimates, only significant paths are included in the path diagram.

Figure 4: Longitudinal stability and cross-lagged effects of depression, anxiety and PTSD on the triple comorbidity of anxiety, depression and PTSD. ***Note:*** All β's are standardized estimates, only significant paths are included in the path diagram.

Figure

Click here to access/download;Figure;Figure 1.tif

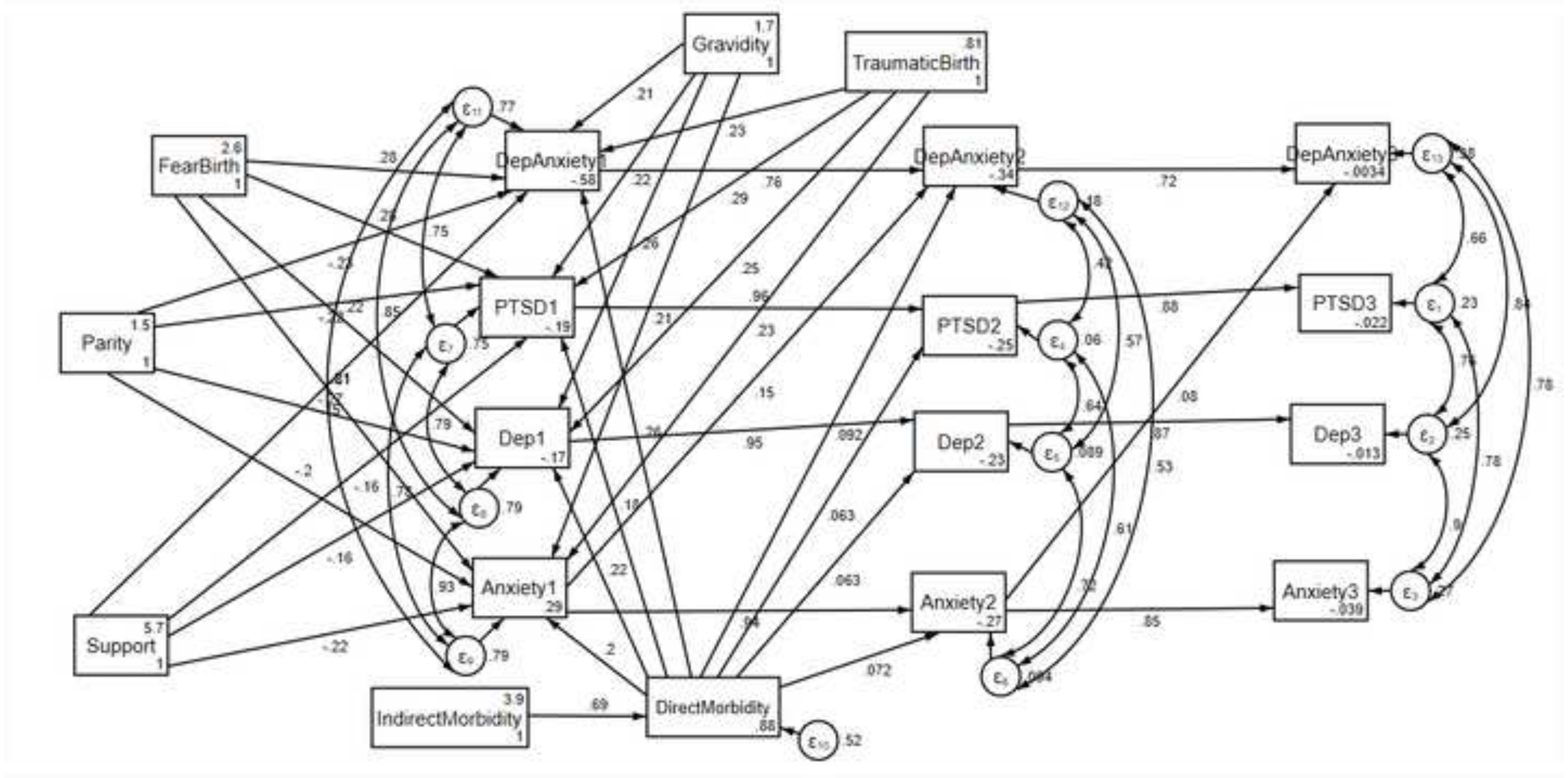

Figure Click here to access/download;Figure;Figure 2.tif

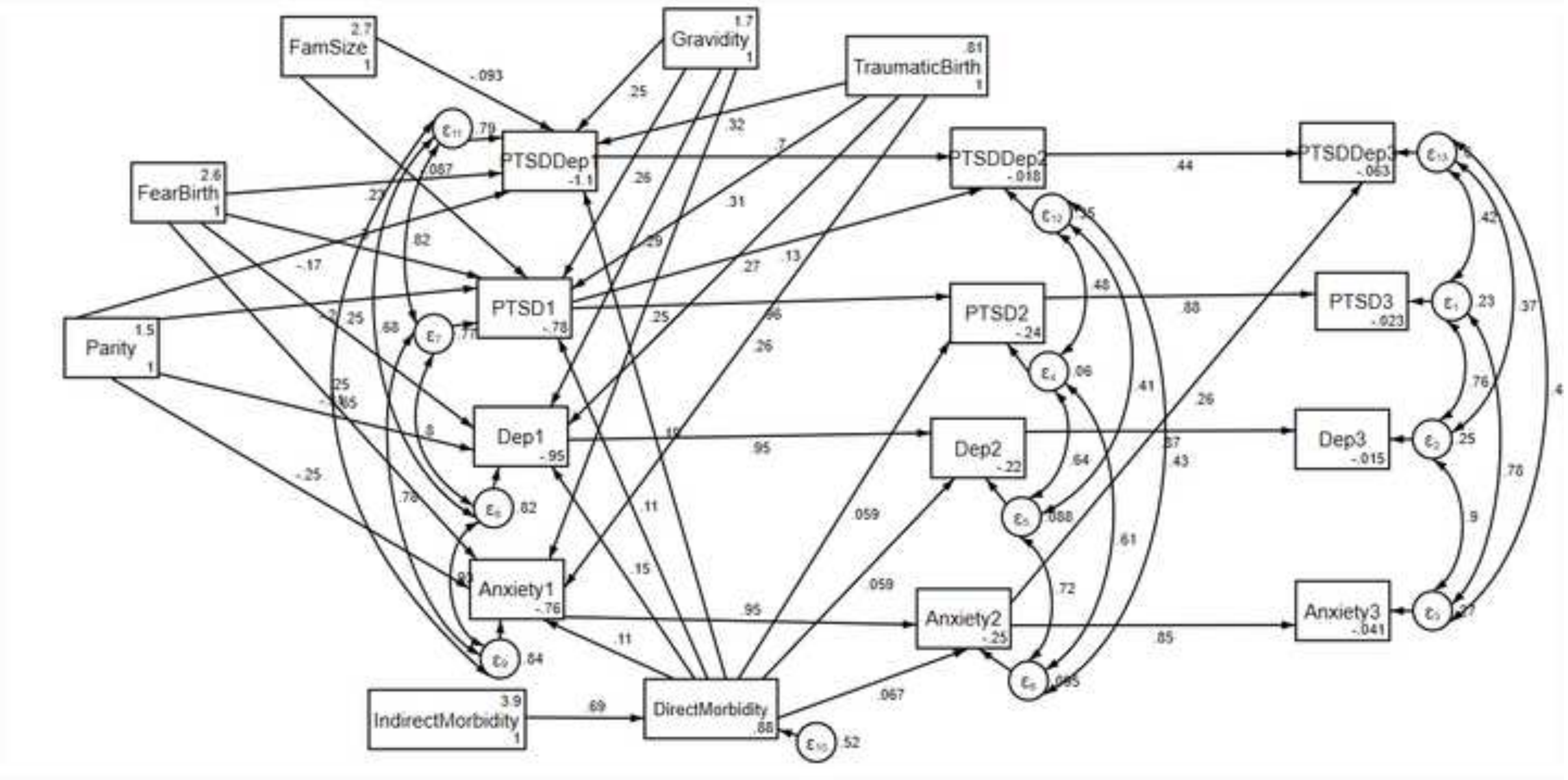

Figure

Click here to access/download;Figure;Figure 3.tif

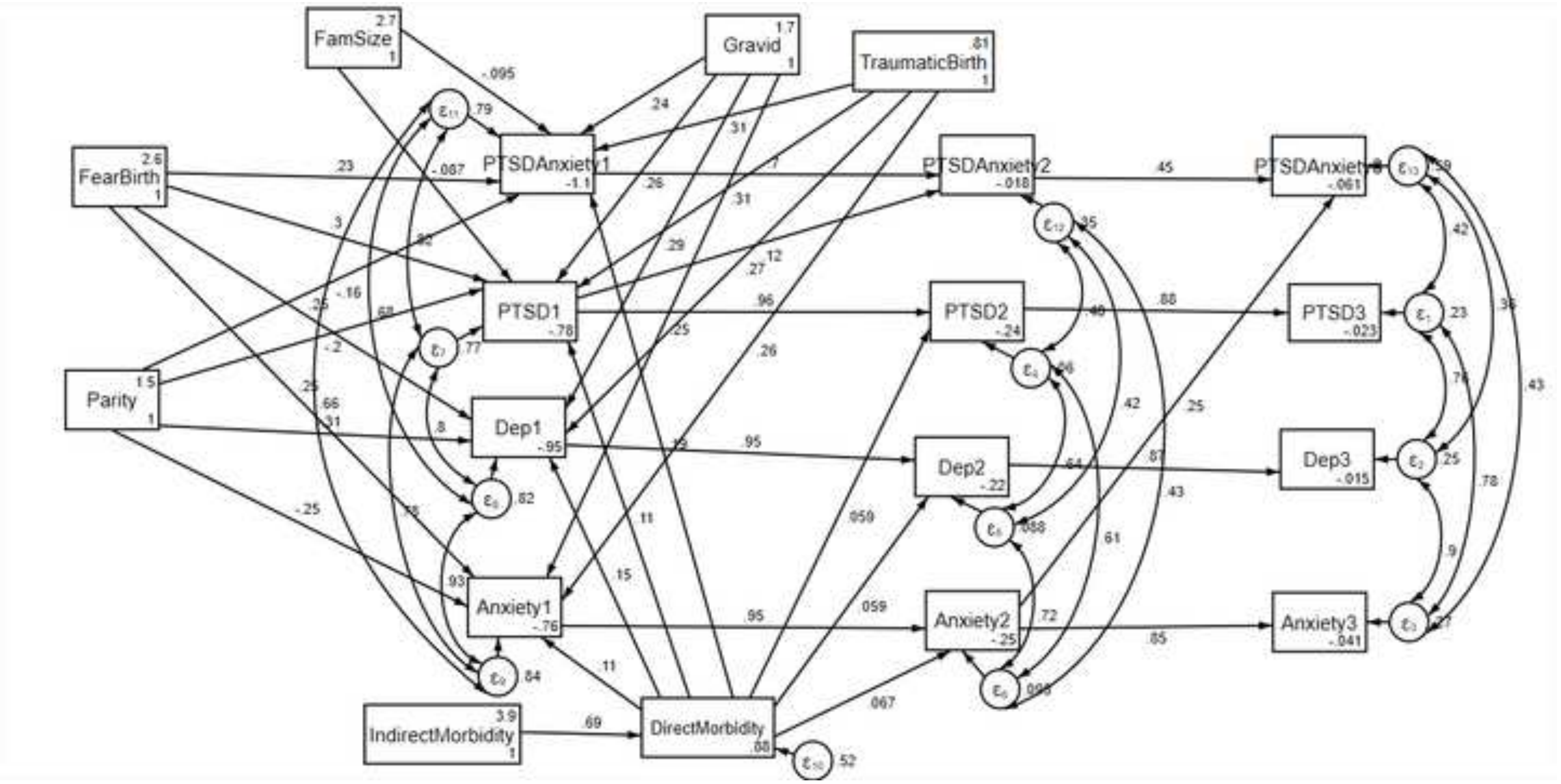

Figure

Click here to access/download;Figure;Figure 4.tif

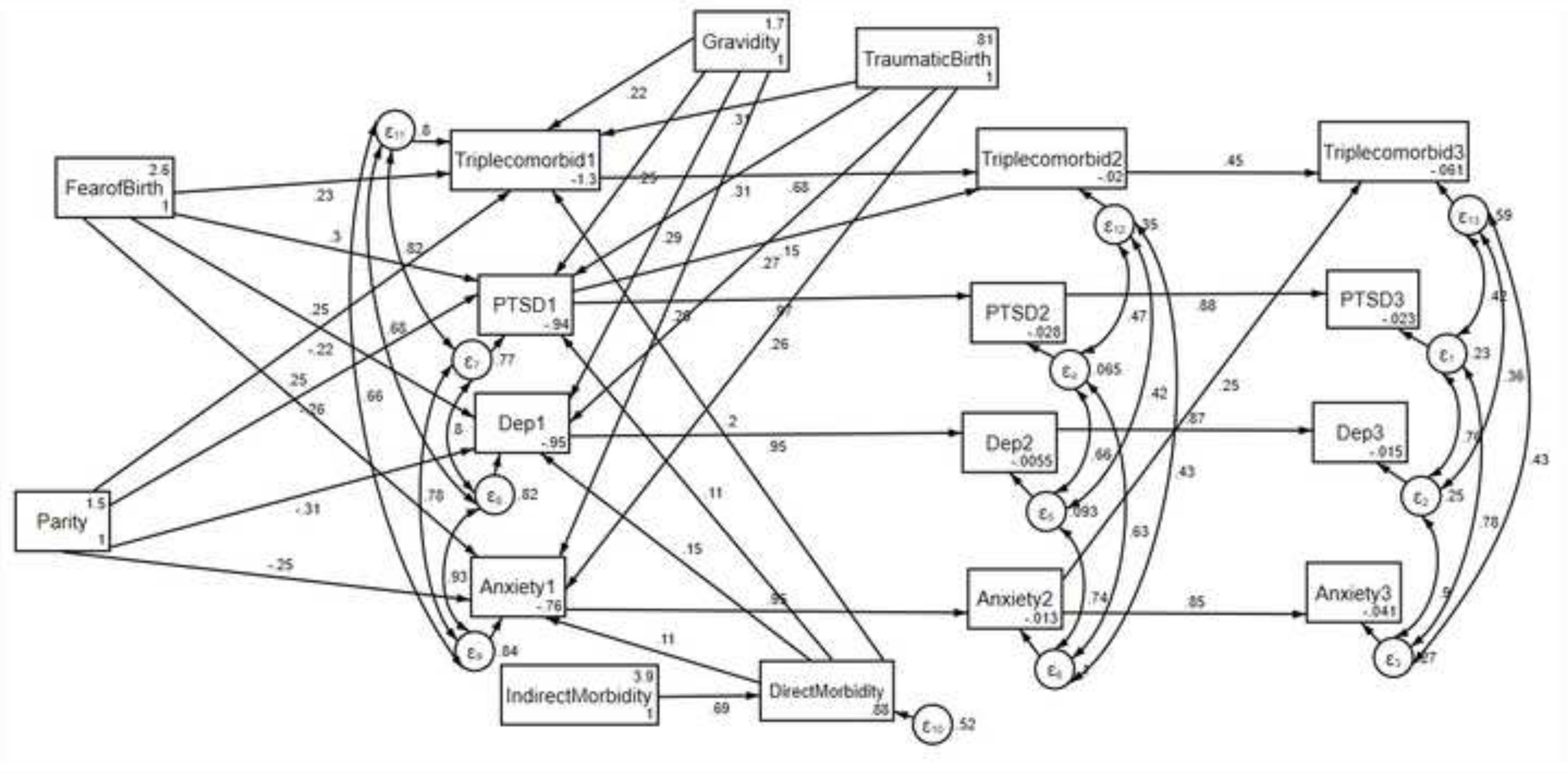

Supplement: Attachment — Submitted filename: PONE-D-22-04381 maternal morbidity path analysis 14052022.pdf [file pone.0273176.s002.pdf]
